# Shared IGHV1-69-encoded neutralizing antibodies contribute to the emergence of L452R substitution in SARS-CoV-2 variants

**DOI:** 10.1080/22221751.2022.2140611

**Published:** 2022-11-11

**Authors:** Qihong Yan, Ruitian Hou, Xiaohan Huang, Yanjun Zhang, Ping He, Yudi Zhang, Banghui Liu, Qian Wang, Haiyue Rao, Xianying Chen, Xinwei Zhao, Xuefeng Niu, Jincun Zhao, Xiaoli Xiong, Ling Chen

**Affiliations:** a State Key Laboratory of Respiratory Disease, Guangdong Laboratory of Computational Biomedicine, Guangzhou Institutes of Biomedicine and Health, Chinese Academy of Sciences, Guangzhou, People’s Republic of China; b State Key Laboratory of Respiratory Disease, Guangzhou Institute of Respiratory Health, the First Affiliated Hospital of Guangzhou Medical University, Guangzhou, People’s Republic of China; c Savaid Medical School, University of Chinese Academy of Science, Beijing, People’s Republic of China

**Keywords:** SARS-CoV-2, L452R, IGHV1-69, Shared antibody response, Immune evasion

## Abstract

SARS-CoV-2 variants continue to emerge facing established herd immunity. L452R, previously featured in the Delta variant, quickly emerged in Omicron subvariants, including BA.4/BA.5, implying a continued selection pressure on this residue. The underlying links between spike mutations and their selective pressures remain incompletely understood. Here, by analyzing 221 structurally characterized antibodies, we found that IGHV1-69-encoded antibodies preferentially contact L452 using germline-encoded hydrophobic residues at the tip of HCDR2 loop. Whereas somatic hypermutations or VDJ rearrangements are required to acquire L452-contacting hydrophobic residues for non-IGHV1-69 encoded antibodies. Antibody repertoire analysis revealed that IGHV1-69 L452-contacting antibody lineages are commonly induced among COVID-19 convalescents but non-IGHV1-69 encoded antibodies exhibit limited prevalence. In addition, we experimentally demonstrated that L452R renders most published IGHV1-69 antibodies ineffective. Furthermore, we found that IGHV1-69 L452-contacting antibodies are enriched in convalescents experienced Omicron BA.1 (without L452R) breakthrough infections but rarely found in Delta (with L452R) breakthrough infections. Taken together, these findings support that IGHV1-69 population antibodies contribute to selection pressure for L452 substitution. This study thus provides a better understanding of SARS-CoV-2 variant genesis and immune evasion.

## Introduction

The coronavirus disease 2019 (COVID-19) pandemic, caused by severe acute respiratory syndrome coronavirus 2 (SARS-CoV-2), is a global health concern. Since the beginning of SARS-CoV-2 pandemic, a number of Variants of Concern (VOCs) or Variants of Interest (VOIs) have emerged, including B.1.1.7 (Alpha), B.1.351 (Beta), P.1 (Gamma), B.1.617.2 (Delta), B.1.429 (Epsilon), and BA.1.1.529 (Omicron BA.1) [1]. Analysis of their spike sequences identified that K417, L452, E484 and N501 within the receptor binding domain (RBD) are mutation hotspots and numerous substitutions have been observed at these locations among variants. It has been proposed that these recurrent RBD substitutions are associated with modulating ACE2-spike interactions and/or evasion of cellular/antibody mediated immunity. These substitutions presumably gave mutant viruses advantages in human-to-human transmission leading to their spread [2,3].

Before global dominance of Omicron BA.1 variant, Delta variant featuring a L452R substitution was the dominant strain worldwide. L452R was first observed in B.1.427/B.1.429 variants which were dominant in California between November and December 2020 [4]. Studies on Delta variant found that L452R mutation reduced neutralizing activities of 14 out of 34 isolated RBD-specific monoclonal antibodies (mAbs). It has milder effect in reducing neutralizing activity of convalescent sera [2,5,6]. Most recently, L452Q, L452M, and L452R have been identified in Omicron subvariants BA.2.12.1, BA.2.13, and BA.4/BA.5, respectively [7], indicating active substitution events at this site. It is currently understood that although mutations are result of error-prone viral replication, dominant variants were selected for transmission advantages. It is conceivable that population immune pressure established from prior infections or vaccinations plays crucial roles in driving virus evolution [8], and variants with ability to evade population antibody responses would possess a considerable transmission advantage.

To help understanding virus evolution, it is therefore important to analyze the prevalence of antibody lineages that are shared among the human population. Variants of SARS-CoV-2 bear mutations that allow escape from neutralization by different classes of antibodies, especially those belonging to classes widely distributed in the human population [9]. Previously, we and others have reported that IGHV3-53-encoded neutralizing antibodies are commonly elicited by SARS-CoV-2 infection and they share similar binding mode to RBD [10–12]. It has been identified that recurrent mutation K417N abolishes neutralization by this class of shared antibodies [12]. Similar to K417N, L452R has been also recurrent in various VoCs. However, it is unclear whether the appearance of L452 substitution is related to the immune pressure of share neutralizing antibodies induced by SARS-CoV-2. In this report, we identified that IGHV1-69 gene encodes antibodies of 3 major clonotypes and these antibodies are widely induced in vaccinees or convalescents of the ancestral SARS-CoV-2 strain (wildtype, WT). We identified that IGHV1-69 antibodies preferentially recognize L452 using preexisting hydrophobic residues at the tip of heavy chain complementary determining region 2 (HCDR2) of germline IGHV1-69 gene. By antigen binding and virus neutralization experiments, we confirmed that L452R can specifically evade binding and neutralization by many IGHV1-69 antibodies. We provide evidence that IGHV1-69-encoded L452-contacting mAbs are differentially recalled in Delta and Omicron BA.1 breakthrough infections, further revealing L452R’s ability to impair immune memory recall in addition to evasion of physical antibody binding. These findings point to an immune selection pressure for L452R mutation contributed by IGHV1-69-encoded neutralizing antibodies. The findings reported here facilitate better understanding of SARS-CoV-2 variant genesis and will help rationalize consequences of immune evasion by generated variants.

## Materials and methods

### Cell lines

HEK293F cells (ATCC, USA) were cultured in CD 293 TGE medium (ACRO, China) with shaking (120–130 rpm) at 37°C in a humidified 5% CO2 incubator. Vero E6 cells (ATCC, USA), 293 T cells (ATCC, USA), and Huh-7 cell (ATCC, USA) were grown in Dulbecco’s modified Eagle’s medium (DMEM) (GIBCO, USA) supplemented with 10% fetal bovine serum (GIBCO, USA) at 37°C in a humidified 5% CO2 incubator.

### Analysis of SARS-CoV-2 spike variants and published SARS-CoV-2 RBD antibody sequences

Per-site variation of SARS-CoV-2 spike was calculated by sequence entropy using the COVID-19 Viral Genome Analysis Pipeline (https://cov.lanl.gov/components/sequence/COV/int_sites_tbls.comp?t=2) that enabled by data from the Global Initiative for Sharing Avian Influenza Data (GISAID; https://gisaid.org). A total of 2438500 SARS-CoV-2 sequences collected before 16 June 2022 were included in this analysis. The germline usage distribution of RBD-targeting mAbs was calculated using the data from COV-AbDab database (the coronavirus antibody database) (http://opig.stats.ox.ac.uk/webapps/covabdab/). A total of 4002 RBD-targeting mAbs isolated from ancestral SARS-CoV-2 infected individuals or vaccinees (accessed before June 22, 2022) were included in this analysis. The frequency distribution of IGHV genes in RBD-targeting mAbs were compared to the IGHV distributions in healthy human antibody repertoires (PRJCA007067 and PRJCA00375) [10,13,14].

### Characteristics analysis of RBD-specific mabs isolated from delta and omicron breakthrough infections

RBD-targeting mAbs isolated from Delta and Omicron BA.1 breakthrough infections were curated from recently published data [15–18]. 110 and 309 mAbs isolated from convalescents experienced Delta breakthrough infections and Omicron BA.1 breakthrough infections were obtained respectively. The frequency distribution of IGHV genes in RBD-targeting mAbs isolated from convalescents experienced Delta and Omicron BA.1 breakthrough infections were compared to the IGHV distributions in mAbs isolated from COVID-19 vaccinees. The HCDR3 amino acids similarity between structurally characterized IGHV1-69-encoded L452-contacting mAbs and IGHV1-69-encoded mAbs isolated from COVID-19 vaccinees, Delta breakthrough infected individuals, or Omicron BA.1 breakthrough infected individuals were calculated using R package stringdist v0.9.8 (https://github.com/markvanderloo/stringdist). Antibody with a GYSGYG/D-like motif or with >75% HCDR3 amino acid similarity to R1-32/FC08 was defined as a R1-32-like antibody [19]. The binding affinity data of R1-32-like antibodies to Delta RBD and WT RBD were compiled from published literatures [16,19–23], and foldchange of the binding affinities of R1-32-like mAbs to Delta RBD relative to WT RBD were calculated.

### Structural analysis

Human SARS-CoV-2 RBD-specific mAbs with available structures were downloaded from the PDB (https://www.rcsb.org/). A total of 221 antibody-RBD/Spike complexes were curated. Buried surface area (BSA) was calculated using the PDBe PISA server (https://www.ebi.ac.uk/msd-srv/prot_int/). Antibody with a non-zero BSA on L452 were identified as a L452-contacting antibody. Structure figures were generated by PyMOL Molecular Graphics System (Schrödinger).

### Analysis of antibody lineage occurrence among L452-contacting antibodies in COVID-19 patients

To determine the occurrence of L452-contacting antibody lineages in COVID-19 patients, we analyzed the IgH sequences from previously published datasets (PRJCA007067 and PRJCA00375) [10,13,14], including 65 blood samples from 33 COVID-19 patients. The V(D)J assignments and HCDR determination were done using MIXCR v3.0.3 [24]. IgH sequences that related to L452-contacting mAbs were defined as those sequences utilizing the same IGHV and IGHJ gene segments, identical HCDR3 length, and ≥75% HCDR3 identity. Visualizations were performed using R software (https://www.r-project.org/). Three IGHV1-69-emcoded L452-contacting antibody clonotypes were identified. Sequence logo plot of the HCDR3s of three L452-contacting antibody clonotypes were visualized using online server Weblogo (https://weblogo.berkeley.edu/logo.cgi).

### Expression of monoclonal antibody

The antibody heavy- and light-V genes (VH/VL) were synthesized (Genscript, China) and were cloned into human IgG1 expression vectors using Clone Express II One Step Cloning Kit (Vazyme, China). When density of HEK293F cells reached 1 × 10^6^ cells/mL, equal amounts of heavy- and light-chain plasmids were transfected into HEK293F cells using EZ cell transfection reagent (Life-iLab Biotech, China). Following transfection, HEK293F cells were cultured in CD 293 TGE medium (ACRO, China) containing 10% CD Feed X supplement (ACRO, China) at 37°C in a humidified 5% CO_2_ incubator shaking at 120 rpm. 6 days post transfection, supernatants were harvested and clarified by centrifugation. Supernatants were filtered through 0.22-µm filters (Merck Millipore, Germany) before incubated with Protein A Resin (Genscript, China) at room temperature for 2 h for antibody affinity purification. After washing, antibodies were eluted from the Protein A Resin using 0.1 M Na-Citrate (pH 3.25) and eluents were neutralized immediately with 1 M Tris-HCl (pH 8.8). Antibodies were concentrated in PBS using Amicon Ultrafilter (Merck Millipore, USA) (GIBCO, USA) and stored at −80°C.

### SARS-CoV-2 RBD expression

HEK293F cells were transfected with plasmids (pcDNA3.1 vector) coding SARS-CoV-2 WT-RBD, L452R-RBD, E484K-RBD, E484Q-RBD, or E484A-RBD (residues 319-541) with a C-terminal 6-His-tag using EZ Cell Transfection reagent (Life-iLab, China). 24 h post transfection, Feed X Supplement was added to culture media at 10% volume ratio. 5 days post transfection, supernatants of cell culture were collected and cleared using 0.22-µm filters. Individual harvested supernatant was supplemented with 25 mM phosphate pH 8.0, 300 mM NaCl, 5 mM imidazole, and 0.5 mM PMSF before recirculated onto a HiTrap TALON crude column (Cytiva, USA) 3 times. The column was washed with buffer A (25 mM phosphate pH 8.0, 300 mM NaCl and 5 mM imidazole), bound RBD was eluted with buffer B (25 mM phosphate pH 8.0, 300 mM NaCl and 500 mM imidazole). Purified RBDs were concentrated in PBS using Amicon Ultrafilters (Merck Millipore, USA) and stored at −80°C.

### Biolayer interferometry

Binding between SARS-CoV-2 RBDs and antibodies was measured by biolayer interferometry (BLI) using an Octet RED96e system (FortéBio, USA). All experiments were performed at 25°C with shaking at 1,000 r.p.m, and anti-human IgG biosensors were pre-equilibrated in Q buffer containing PBS (10 mM pH7.4), 0.02% Tween-20, and 0.2% BSA for at least 600 s before use. Antibodies were diluted to 11 μg/mL before loaded onto protein A biosensors for 60 s. Antibody loaded biosensors were dipped into wells containing 200 nM SARS-CoV-2 RBD-His recombinant proteins or Q buffer (as control) for 300 s to monitor association. Dissociation was monitored by moving biosensors into Q buffer for 300 s. Binding data was analyzed using FortéBio data analysis software.

### ELISA assay to determine antibody binding activity to SARS-CoV-2 RBD

96 well assay plates were coated with 100 µL per well of SARS-CoV-2 WT-RBD, E484K-RBD, E484Q-RBD, or E484A-RBD recombinant proteins at 1 µg/mL in PBS overnight at 4°C, respectively. After standard washing, 5% skim milk (200 µL per well) was added to block for 2 h at 37°C. After washing wells three times with PBS-0.05% Tween 20 (MP), 100 µL semilogarithmic dilutions in PBS of antibodies were added to each well and incubated at 37°C for 2 h. After washing wells 3 times with PBS-0.05% Tween 20, plates were incubated with 1:5000 dilutions of HRP-labelled Goat Anti-Human IgG (H + L) (Beyotime, China) in 5% skim milk at 37°C for 1 h. After washing wells 6 times with PBS-0.05% Tween 20, 100 µL per well of TMB/E solution (Merck Millipore) was added and developed at room temperature for 15 min. Reactions were stopped by adding 50 µL 1 M sulphuric acid and OD values at 450 nm were measured.

### SARS-CoV-2 foci reduction neutralization test

Antibodies were serial diluted with DMEM and mixed with 200 FFU Wuhan-Hu-1 (wildtype) or Delta variant authentic SARS-CoV-2 viruses. After incubation at 37°C for 1 h, antibody-virus mixtures were added to a 96-well plate cultured with Vero E6 cells and incubated at 37°C in 5% CO_2_ for 1 h. After removing the inocula, plates were overlaid with 100 μL 1.6% carboxymethylcellulose warmed to 37°C per well After culturing for 24 h, overlays were removed and the cells were fixed with 4% paraformaldehyde (Biosharp, China) and permeabilized with 0.2% Triton X-100 (Sigma, USA). Cells were incubated with a human anti-SARS-CoV-2 nucleocapsid protein monoclonal antibody (obtained by laboratory screening) at 37°C for 1 h. After washing with 0.15% PBST three times, cells were incubated with an HRP-labeled goat anti-human secondary antibody (Cat. No.: 609-035-213, Jackson ImmunoResearch Laboratories, Inc. West Grove, PA) at 37°C for 1 h. Plates were washed with 0.15% PBST three times, before the foci were visualized by TrueBlue Peroxidase Substrate (KPL, Gaithersburg, MD), and counted with an ELISPOT reader (Cellular Technology Ltd. Cleveland, OH). The foci reduction neutralization test (FRNT50) was calculated by Spearman-Karber method. The SARS-CoV-2 WT was isolated from a COVID-19 patient [25]. This virus is stored in Guangzhou Customs District Technology Center BSL-3 Laboratory. The SARS-CoV-2 Delta (B.1.617.2) strain is stored at Guangdong Provincial Center for Disease Control and Prevention, China. Experiments using SARS-CoV-2 authentic viruses were conducted in Guangzhou Customs District Technology Center BSL-3 Laboratory.

### Pseudovirus-neutralization assay

SARS-CoV-2 WT, Omicron BA.1.1.529, BA.2.12.1, BA.2.13, and BA.4/BA.5 spike plasmids were constructed using the pcDNA3.1 vector. G*ΔG-VSV virus (VSV G pseudotyped virus) was used to infect 293T cells, and spike protein-expressing plasmid was used for transfection at the same time. After culture, the supernatant containing pseudovirus was collected, filtered, aliquoted, and frozen at −80 °C for further use. Monoclonal antibodies were serially diluted (threefold) in DMEM (GIBCO, USA) and mixed with pseudovirus in 96-well plates. After incubation at 5% CO2 and 37 °C for 1 h, digested Huh-7 cells were seeded. After 24 h of culture, supernatant was discarded and d-luciferin reagent (PerkinElmer, USA) was added to react in the dark. The luminescence value was measured using a microplate spectrophotometer (PerkinElmer, USA). IC50 was calculated by a four-parameter logistic regression model using PRISM v 8.0.1.

## Results

### L452-contacting mAbs show IGHV germline gene preference

Analysis of 2,438,500 SARS-CoV-2 sequences deposited in GISAID database (https://www.gisaid.org/) showed that L452 is one of the most active mutation hotspot on RBD (Figure S1A). Structural analysis showed that L452 is in proximity to the RBD-ACE2 binding interface (Figure S1B), but pointing away from the interface to form a hydrophobic patch with F490 (we noted 490 is also a mutation hotspot) and L492 (Figure S1C). Substitution of L452 to a charged residue R led to a moderate increase in affinity towards ACE2 receptor (Figure S1C,D), increased ACE2 affinity was shown to enhance infectivity of human cells [5].

**Figure 1.**
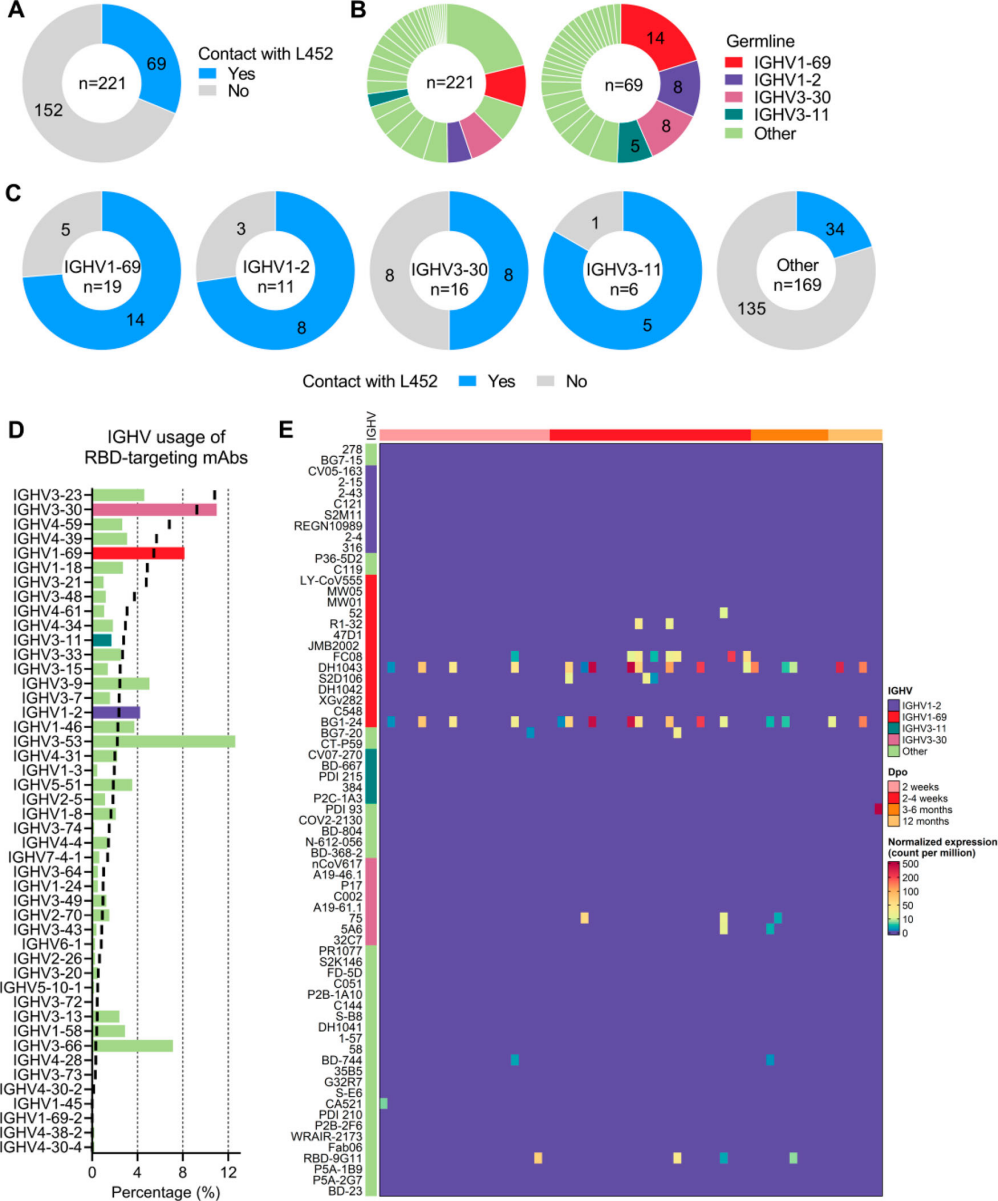
Characteristics of human mAbs contacting L452 in SARS-CoV-2 spike. (A) Pie chart showing the proportion of mAbs contacting L452 within the PDB. (B) Pie chart showing the germline distribution of 221 RBD-targeting mAbs (left panel) and 69 mAbs that contact with L452 (right panel). (C) Pie chart showing the proportion of mAbs that contact with L452 among IGHV1-69, IGHV1-2, IGHV3-30, IGHV3-11, or other IGHVs-encoded RBD-specific mAbs that are deposited in the PDB. (D) IGHV germline gene usage by RBD-targeting mAbs compared to antibody repertoires of healthy individuals. Histogram represents the IGHV germline gene usage of RBD-targeting mAbs, black lines represent the IGHV germline gene usage in antibody repertoires of healthy individuals [10,13,14]. (E) Heatmap showing the occurrences of L452-contacting mAbs in blood samples collected at different timepoints of 33 COVID-19 convalescents [10,13,14]. The top annotation represents the sample collection time (Dpo: Days post symptom onset). The left annotation represents germline IGHV genes of L452-contacting mAbs. Colour bar represents the abundance of IgH sequences that related to L452-contacting mAbs.

To identify neutralizing antibodies contacting L452, we analyzed epitopes of all the human mAbs structures in complex with SARS-CoV-2 RBD/Spike available in the Protein Data Bank (PDB) (deposited before 22 June, 2022) (Table S1). We identified that 69 out of 221 human RBD-targeting mAbs contact with residue L452 (Figure 1(A)), including those approved for emergency use authorization (EUA) or in clinical trials such as LY-CoV555 (bamlanivimab), CT-P59 (regdanvimab), COV2-2130 (cilgavimab), and REGN10933 (casirivimab) (Table S1). Although the 69 L452-contacting mAbs are derived from 29 different IGHV genes, more than half of them are encoded by four IGHVs: IGHV1-69, IGHV1-2, IGHV3-30, and IGHV3-11 (Figure 1(B)). We investigated percentage of L452-contacting antibodies among the structurally characterized antibodies encoded by the four IGHV genes. We found that 14/19, 8/11, 8/16, and 5/6 of structurally characterized IGHV1-69, IGHV1-2, IGHV3-30, and IGHV3-11 antibodies contact L452, respectively (Figure 1(C)). This observation suggests potential preferential germline usage for L452 recognition. Germline gene usage analysis of 4002 isolated RBD-targeting mAbs from CoV-AbDab (the coronavirus antibody database) [26] (deposited before June 22, 2022) showed that IGHV1-69, IGHV3-30, IGHV1-2 are over-represented in this database. In particular, IGHV3-30 and IGHV1-69 ranked 2nd and 3^rd^ among isolated RBD-targeting mAbs. Of note, it has been previously shown that IGHV3-30 and IGHV1-69 are among the most frequently utilized antibody genes, ranking 2nd and 5th among healthy human antibody repertoires (Figure 1(D)).

### IGHV1-69-encoded L452-contacting antibodies are widely induced in COVID-19 patients

To determine the occurrence of L452-contacting antibodies induced by SARS-CoV-2 infection, we attempted to identify their related antibody lineages from antibody repertoire data of COVID-19 patients we previously sequenced. Antibody repertoire data from blood samples collected at various time points after infection in a cohort of 33 COVID-19 patients were used [10,13,14]. We were able to identify antibody sequences highly similar (heavy chain with the same IGHV and IGHJ, identical HCDR3 length, and ≥75% HCDR3 AA sequence identity) to the structurally characterized IGVH1-69-encoded L452-contacting mAbs. Three highly shared antibody clonotypes encoded by IGHV1-69 are widely present in the antibody repertoires of COVID-19 patients (Figure 1(E), Figure S2A). The first clonotype is related to the L452-targeting antibody S2D106, whose HCDR3s share a conserved CSGGSCY motif. The second clonotype is related to the L452-contacting antibodies DH1043 and BG1-24, whose HCDR3s share a conserved YYDSSGY motif. The third clonotype is related to our previously identified R1-32-like antibodies that share a GYSGYG/D motif in their HCDR3s [19] (Figure S2A). Whereas sequences similar to structurally characterized IGHV1-2, IGHV3-30, IGHV3-11 and other IGHVs encoded L452-contacting mAbs showed no or limited detection in COVID-19 patients (Figure 1(E)). Given that sequences highly similar to IGHV1-69-encoded RBD-targeting neutralizing antibodies were widely identified in a broad COVID-19 patient population, we speculate IGHV1-69-encoded RBD-targeting antibodies are likely involved in SARS-CoV-2 herd immunity, and L452R substitution is likely evolved to counteract IGHV1-69-encoded neutralizing antibodies shared among population.

### IGHV1-69-encoded L452-contacting mAbs possess a germline hydrophobic HCDR2 loop for L452 recognition

**Figure 2.**
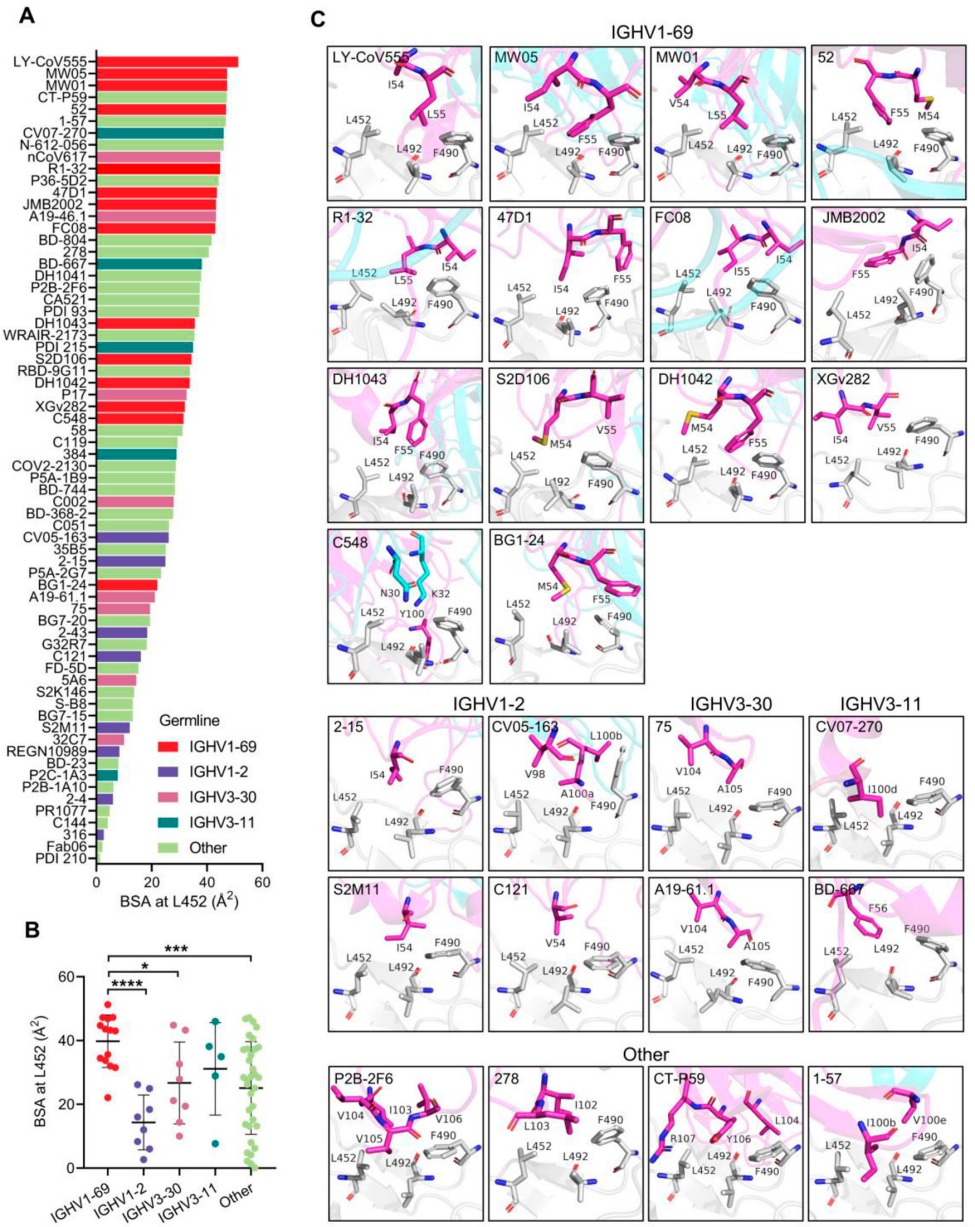
Structural analysis of the L452-contacting mAbs. (A) Histogram showing the buried surface area (BSA) at L452 by L452-contacting mAbs. (B) Comparison of buried surface areas at L452 among IGHV1-69, IGHV1-2, IGHV3-30, IGHV3-11, and other IGHVs-encoded L452-contacting mAbs. ****p<0.0001, ***p<0.001, **p<0.01, *p<0.05 by unpaired t test. (C). Binding mode comparison of L452-contacting mAbs encoded by IGHV1-69, IGHV1-2, IGHV3-30, IGHV3-11, and other IGHV genes. The residues involved in the interactions are shown as sticks. Residues in the RBD are coloured in grey, and residues in antibody heavy chain and light chains are coloured in magenta and cyan, respectively.

We further investigated structurally characterized antibodies and their interactions with L452 by computing buried surface areas (BSAs) at 452. We found that IGHV1-69-encoded mAbs generally bury more surface area at 452, followed by IGHV3-11, IGHV3-30, and IGHV1-2 (Figure 2(A)). Of note, the EUA mAb LY-CoV555 has the highest BSA at L452 and another EUA mAb CT-P59 ranks 4^th^ (Figure 2(A)). Both antibodies have been shown to be escaped by the Delta variant [2]. The mean BSA for residue L452 in IGHV1-69-encoded mAbs is 39.8±8.2 Å^2^, by comparison, the mean BSA for residue L452 in IGHV1-2, IGHV3-30, IGHV3-11, and other IGHVs-encoded mAbs is 14.3±8.6 Å^2^, 25.9±11.7 Å^2^, 31.2±14.5 Å^2^, and 25.1±14.5 Å^2^, respectively (Figure 2(B)). This result suggests IGHV1-69-encoded mAbs likely have a preference for L452 recognition. Next, we compared binding modes of the L452-contacting mAbs. The 14 IGHV1-69-encoded mAbs segregated into two groups, one comprised of mAbs (LY-CoV555, MW01, MW05, DH1043, S2D106, DH1042, BG1-24, C548, and XGv282) that targeted an ACE2-competitive site and the other containing mAbs (47D11, JMB2002, FC08, R1-32, and 52) that recognized a non-ACE2 competitive region (Figure S2B). We found that most ACE2-competitive IGHV1-69-encoded mAbs share similar binding modes approaching RBD with similar angles. Non-ACE2-competitive mAbs FC08, R1-32, and 52 also share similar binding mode. The conserved motifs in HCDR3s of the different clonotype appear to play a key role for RBD engagement and probably determined antibody binding mode (Figure S2C). However, importantly, we identified that whether they are ACE2-competitive or not these IGHV1-69-encoded mAbs share a common feature in L452 recognition (Figure 2(C), Figure S2B). Except for mAb C548, all the other 13 IGHV1-69-encoded mAbs use HCDR2 hydrophobic residues to interact with the L452 formed hydrophobic patch (Figure 2(C)). Of note, these hydrophobic residues represent a conserved feature on the tip of IGHV1-69 HCDR2 loop (positions 54, 55) and are encoded by IGHV1-69 germline alleles (Figure 2(C), Figure S3A). In contrast, no conserved hydrophobic residue was observed for IGHV1-2, IGHV3-30, IGHV3-11 or other IGHVs encoded mAbs to interact with L452, albeit hydrophobic contact serves as the most common mode of L452 interaction. Non-IGHV1-69 antibodies contact L452 by somatically mutated residues (e.g. N54I (2-15, S2M11), N54V (C121), and S56F (BD-667)) (Figure 2(C)) or by HCDR3 residues generated from VDJ recombination (e.g. V98 (CV05-163), V104 (75, A19-46.1, P2B-2F6), Y106 (CT-P59), L103 (278), and I100b (1-57)) (Figure 2(C)). Even though some somatic mutations were observed in HCDR2 loops of the characterized IGHV1-69-encoded antibodies including I54V (MW01), I54M (BG1-24, S2D106, 52, DH1042), L55I (FC08), and L55V (XGv282), all these were mutations between hydrophobic amino acids (Figure S3(A,B)). Among the 69 structurally characterized L452-contacting mAbs, only the IGHV1-69-encoded mAbs possess multiple hydrophobic residues at the tip of HCDR2 loop. Hydrophobicity analysis of all the human IGHV germline genes revealed that the IGHV1-69 (19 alleles) is the only human heavy-chain gene that encodes hydrophobic residues at the tip of HCDR2 loop (Figure S3C), indicating L452 binding can be achieved by germline IGHV1-69 antibodies without requiring somatic hypermutation. Whereas somatic hypermutations or VDJ rearrangements are needed for IGHV1-2, IGHV3-30, IGHV3-11 or other IGHVs-encoded mAbs to acquire hydrophobic residues (Figure S3B).

### IGHV1-69-encoded L452-contacting mAbs are evaded by the L452R substitution

**Figure 3.**
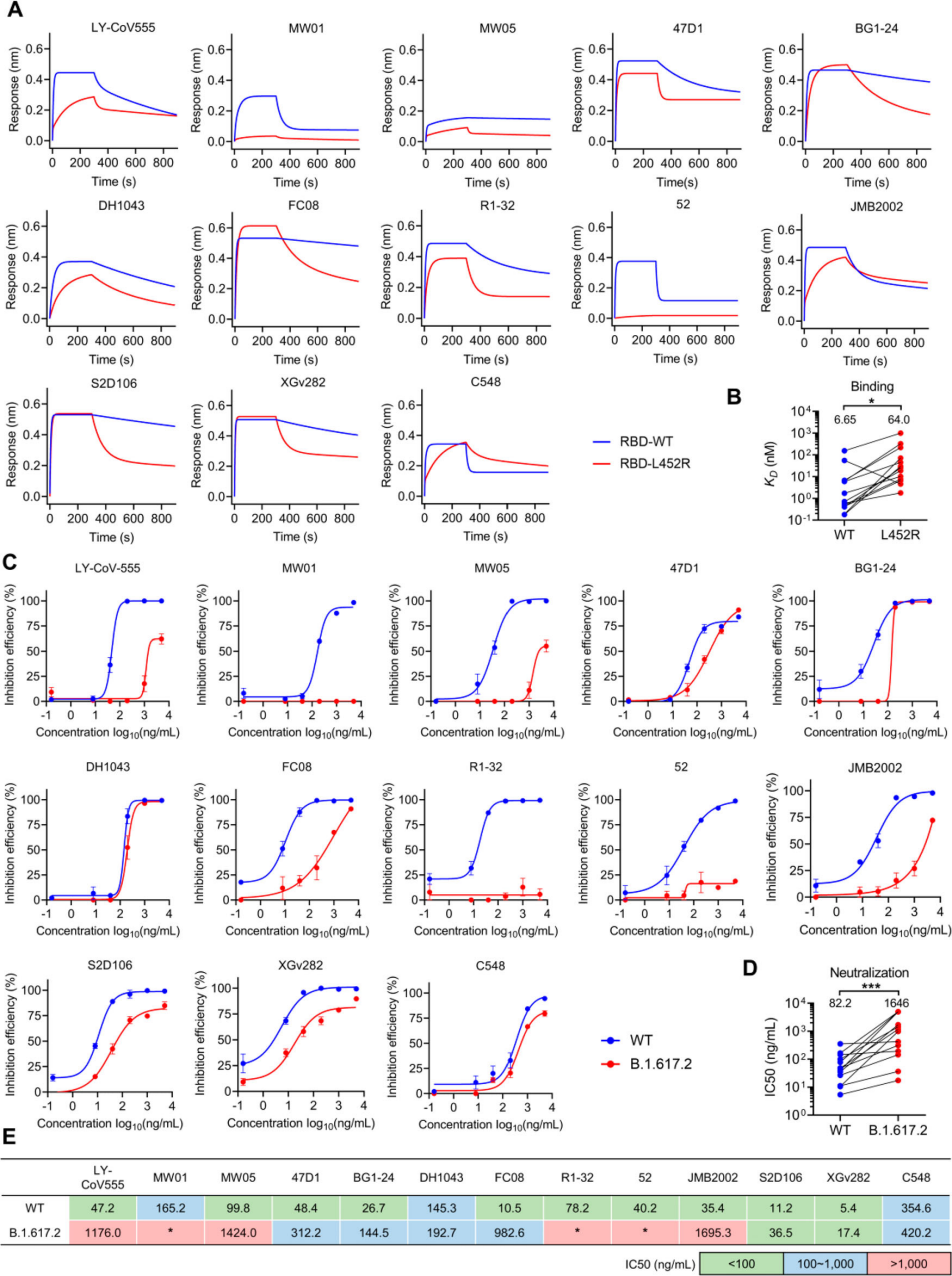
Binding and neutralization activities of IGHV1-69-encoded L452-contacting mAbs. (A) Binding abilities of IGHV1-69-encoded mAbs to wildtype (WT) or L452R RBDs were assessed by BLI. (B) Comparison of binding abilities of IGHV1-69-encoded mAbs to WT and L452R RBDs. The y-axis represents equilibrium dissociation constant (*K_D_*). (C) Neutralizing activities of IGHV1-69-encoded mAbs to WT or B.1.617.2 (Delta) viruses were measured by FRNT. (D) Comparison of the neutralizing abilities of IGHV1-69-encoded mAbs to WT and B.1.617.2 (Delta) authentic viruses. The y-axis represents half maximal inhibitory concentration (IC50). (E) IC50 values of the IGHV1-69-encoded L452-contacting mAbs against WT or B.1.617.2 (Delta) viruses. green, IC50 ≤ 100 ng/mL; blue, 100 ng/mL < IC50 < 1,000 ng/mL; red, IC50 ≥ 1,000 ng/mL; *, IC50 ≥ 5,000 ng/mL. Statistical tests in panel B and D are performed using two-tailed Wilcoxon signed-rank tests of paired samples. *, *p* < 0.05; **, *p* < 0.01, *p* < 0.001, ***.

To test whether L452R substitution confers resistance to the structurally characterized L452-contacting mAbs. We expressed a panel of representative mAbs for binding and neutralization tests. The binding assays showed that almost all the tested IGHV1-69-encoded mAbs (except C548) exhibited reduced affinity to L452R RBD (Figure 3(A)), with an average reduction of 8.6 folds (Figure 3(B)). Meanwhile, we found that mAbs such as nCoV617 (IGHV3-30), CV07-270, BD-667 (IGHV3-11), P2B-2F6, and BD-368-2 that derived from non-IGHV1-69 germlines also can be heavily affected by L452R substitution (Figure S4A). However, we observed some of the L452-contacting mAbs such as 2-15, CV05-163 (IGHV1-2), and A19-61.1 (IGHV3-30) that generally have lower BSAs at L452 (Figure 2(A,B)) are unaffected by L452R substitution (Figure S4A). Further analysis found that the sensitivities of these L452-contacting mAbs to L452R are negatively correlated with their BSAs at L452 (Figure S4B). This observation explains why most IGHV1-69-encoded mAbs are sensitive to L452R because they have a higher BSAs at L452 (Figure 2(A,B)). Consistent with the decreased binding affinities to L452R, the tested IGHV1-69-encoded mAbs also exhibited reduced neutralization of L452R bearing B.1.617.2 variant (Delta) (Figure 3(C)), with an average reduction of 19 folds (Figure 3(D)). Next, we investigated whether these IGHV1-69-encoded mAbs are affected by Omicron (without L452 substitution) or its subvariants (with L452X substitution). By pseudovirus-neutralization assay we showed that ACE2-competitive mAbs, LY-CoV555, MW01, and DH1043, are generally sensitive to both Omicron and its subvariants. However, the non-ACE2-competitive mAbs, R1-32 and FC08, are relatively insensitive to Omicron and BA.2.13 (with L452M) but more sensitive to BA.2.12.1 (with L452Q) and BA.4/BA.5 (L452R) (Figure S4C). Collectively, the above results showed that L452R significantly reduced affinity and neutralization activities of the IGHV1-69-encoded antibodies (Figure 3(B,D)), rendering most of them ineffective (Figure 3(E)).

### IGHV1-69-encoded L452-contacting mAbs are enriched in Omicron BA.1 breakthrough infection but not in Delta breakthrough infection

**Figure 4.**
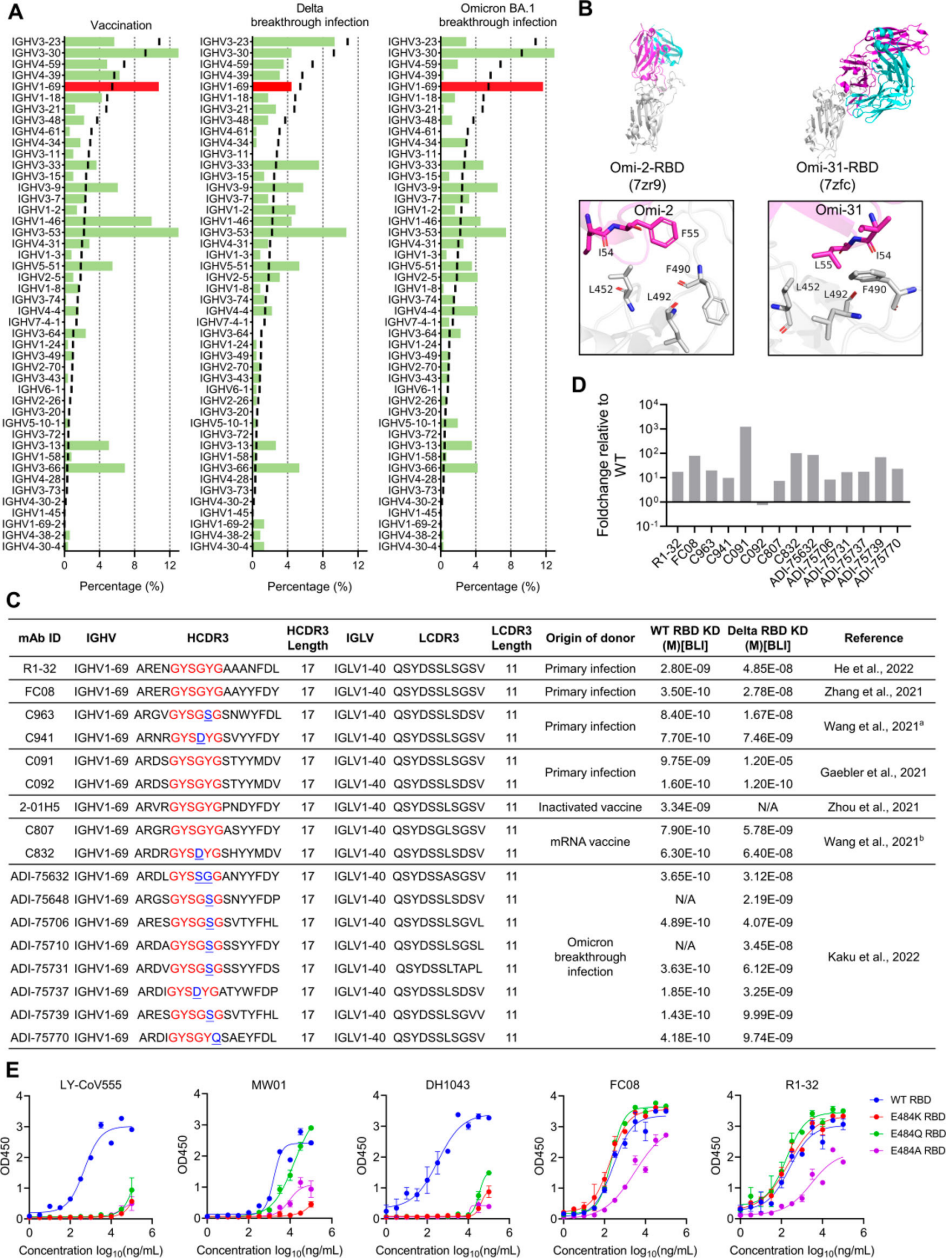
Characteristics and prevalence of IGHV1-69-encoded L452-contacting mAbs among antibodies isolated from vaccinees, Delta or Omicron breakthrough infected individuals. (A) Comparison of IGHV germline gene usage of RBD-targeting mAbs in among antibodies isolated from vaccinees, Delta breakthrough infected, and Omicron breakthrough infected individuals. Black lines represent the IGHV germline gene usage in antibody repertoires of healthy individuals [10,13,14]. (B) Binding mode of L452-contacting mAbs Omi-2 and Omi-31. The residues involved in the interactions are shown as sticks. Residues in the RBD are coloured in grey, and residues in antibody heavy chain and light chains are coloured in magenta and cyan, respectively. (C) Summary of the R1-32-like mAbs isolated from individuals with SARS-CoV-2 primary infections, or receiving inactivated or mRNA vaccines, or with Omicron breakthrough infections [16, 19, 20, 21, 22, 23]. (D) Fold-change of the binding affinities of R1-32-like mAbs to Delta RBD relative to WT RBD. (E) Binding abilities of five IGHV1-69-encoded L452-contacting mAbs to WT, E484K, E484Q, and E484A RBDs as measured by ELISA.

Since L452R substitution confers resistance to IGHV1-69-encoded RBD-targeting mAbs (Figure 3), and IGHV1-69-encoded RBD-targeting mAbs are widely elicited by primary infection of the SARS-CoV-2 ancestral strain (Figure 1(C)), it raises the question whether IGHV1-69-encoded mAbs could be induced by COVID-19 vaccination or breakthrough infection. To answer this question, we first investigated the germline usage of 618 RBD-targeting mAbs isolated from COVID-19 vaccinees in CoV-AbDab [26]. Consistent with primary SARS-CoV-2 infection, IGHV1-69-encoded mAbs are highly enriched in COVID-19 vaccinees similar to IGHV3-53/3-66 and IGHV3-30 mAbs (Figure 4(A)), indicating similar RBD-specific antibody induction between vaccination and natural infection. However, we did not observe an enrichment of IGHV1-69 in Delta breakthrough infection. Only 10 (4.4%) IGHV1-69-encoded mAbs was found among 225 Delta RBD-reactive mAbs [17,18], indicating that IGHV1-69 encoded antibodies did not respond to Delta breakthrough infection (Figure 4(A)). In contrast to IGHV1-69, IGHV3-53/3-66 are over-represented in Delta breakthrough infection because this class of antibodies, including B-38 [27], CC12.1 [11], COVOX-222 [28], and rmAb23 [10], are known to maintain neutralizing activities to Delta variant (Figure S5). Unlike Delta breakthrough infection, Omicron BA.1 (without L452 substitution) breakthrough infection massively induced IGHV1-69-encoded antibody clones, representing with 11.65% (2nd most frequent) of 309 Omicron RBD-reactive mAbs [15,16] (Figure 4(A)). Structural analysis showed that two IGHV1-69-encoded Omicron RBD-reactive mAbs Omi-2 and Omi-31 that isolated from Omicron BA.1 breakthrough infection shared the same binding mode with those isolated from primary SARS-CoV-2 ancestral strain infection (Figure 4(B), Figure 2(C), Figure S2B). Both Omi-2 and Omi-31 use their hydrophobic residues at the tip of HCDR2 loops to engage the L452 containing hydrophobic patch (Figure 4(B)) and Omi-31 is heavily affected by L452R substitution [15].

Sequence analysis showed that IGHV1-69-encoded mAbs isolated from vaccination and Omicron BA.1 breakthrough infection but not from Delta breakthrough infection are highly similar with the structurally characterized L452-contacting mAbs encoded by IGHV1-69 (Figure S6A). Notably, most of the IGHV1-69-encoded clones isolated from Omicron BA.1 breakthrough infection utilize IGLV1-40 light-chain gene and have a preferred length of 17 amino-acid HCDR3s and a GYSGYG/D-like motif, which are related to our previous reported R1-32-like antibody lineage (non-ACE2 competitive) that isolated from primary SARS-CoV-2 ancestral strain infection or vaccination (Figure 4(D), Figure S2A, Figure S6B). By reanalyzing the binding affinity data [16], we found that the IGHV1-69-encoded clones isolated from Omicron BA.1 breakthrough infection showed around 10-fold reduced binding affinity to the Delta variant (Figure 4(C,D)). Structural analysis revealed that the epitope residues of R1-32-like mAbs (R1-32 and FC08) are not affected by mutations in Omicron BA.1, whereas most of ACE2-competitive IGHV1-69 mAbs largely interact with E484 (Figure S6B). We showed by ELISA that ACE2-competitive IGHV1-69 mAbs, LY-CoV555, MW01, and DH1043, are generally sensitive to E484 mutations (Omicron BA.1 has E484A) but the non-ACE2-competitive IGHV1-69 mAbs, R1-32 and FC08, are relatively insensitive to the E484 mutations (Figure 4(E)). The above observations suggest a preferential recalling of the non-ACE2-competitive IGHV1-69-encoded memory B cell response following Omicron BA.1 breakthrough infection, thereby increasing selection for L452.

## Discussion

In summary, we report detection of three shared IGHV1-69-encoded antibody clonotypes in broad population exposed to SARS-CoV-2 antigen. Structural and genetic analysis revealed that germline IGHV1-69 genes encode multiple hydrophobic residues at the tip of HCDR2 loop to allow contact to the L452 formed hydrophobic patch. This binding mode is shared by most characterized IGHV1-69-encoded neutralizing antibodies. Unlike other IGHVs-encoded L452-contacting antibodies, IGHV1-69-encoded antibodies appear to have preexisting ability to bind L452 without requiring VDJ recombination or somatic hypermutation. These unique characteristics and frequent presentation of IGHV1-69-encoded mAbs in human antibody repertoires led us to propose that this class of antibodies contributes to immune pressure driving the L452R mutation. Consistently, we demonstrated experimentally that L452R was able to abrogate binding and neutralization by most known IGHV1-69-encoded L452-contacting mAbs.

In addition to natural SARS-CoV-2 infection, SARS-CoV-2 vaccination also induced the enrichment of L452-contacting mAbs encoded by IGHV1-69, suggesting an established population immune pressure from prior infections or vaccinations. However, the preferential induction of IGHV1-69 was not observed in breakthrough infections by the L452R bearing Delta variant, indicating that L452R substitution not only confer virus ability to evade neutralization by IGHV1-69-encoded mAbs but also ability to suppress reactivation of immune memory. In this respect, IGHV1-69 antibody response is different to IGHV3-53/3-66 responses that even though Omicron variants contain the K417N mutation, IGHV3-53/3-66-encoded antibodies (also a shared antibody response) could be recalled following Omicron BA.1 breakthrough infection with some recalled mAbs showing K417N resistance [15]. These observations confirmed that certain spike mutations may not only evade physical binding by antibodies but also modulate antibody responses by suppressing induction of certain antibody classes.

While preparing this manuscript, we noted that L452 in the current circulating Omicron sub-variants is undergoing continued evolution. L452Q, L452M, and L452R have been identified in BA.2.12.1, BA.2.13, and BA.4/BA.5, respectively. Our analysis and existing report [16] showed that Omicron BA.1 breakthrough infection massively recalled the wildtype-induced non-ACE2 competitive L452-contacting mAbs encoded by IGHV1-69. The biased induction of non-ACE2-competitive IGHV1-69 antibodies especially the R1-32-like antibodies in Omicron BA.1 breakthrough infection likely enhanced selection pressure for L452 substitution. These IGHV1-69 mAbs showed cross-reactivities to both wildtype and Omicron BA.1 viruses, but are heavily escaped by L452R. Consistent with our results, Cao et al., recently found that the FC08 and other R1-32-like IGHV1-69 class of mAbs (e.g. BD-380, BD-421, BD-901, C091, XGv-271) are sensitive to L452X mutations using high-throughput yeast-display-based deep mutational scanning (DMS) assays [7]. In addition, SARS-CoV-2 BA.4/BA.5 escape mAbs and serum antibodies elicited by Omicron BA.1 infection, particularly the newly added mutation L452R and F486V in BA.4/BA.5 [7, 15, 29]. The analysis presented in this report provides some explanation for the rapid emergence of L452 substitutions in Omicron subvariants. Moreover, since Omicron BA.1 breakthrough infection induced a skewed antibody response targeting L452, it likely increased the possibility of repeated infection by L452R harbouring BA.4/BA.5.

Existing studies have shown that certain antibodies against a particular epitope share a restricted set of immunoglobulin heavy chain variable region genes and are often quite similar in overall structure and function [30,31]. For example, broadly neutralizing antibodies targeting the E2 antigenic region 3 (AR3) of HCV are mainly derived from IGHV1-69 [32,33]. IGHV1-69 has been also identified as one of the major classes of antibodies against the Influenza hemagglutinin (HA) stem region [34,35]. In these studies, it has been demonstrated that IGHV1-69 germline-encoded HCDR2 hydrophobic residues are essential for HCV and Influenza epitope recognition [31], consistent with our finding on IGHV1-69-encoded SARS-CoV-2 S-specific antibodies. Therefore, a recurring theme emerges that hydrophobic epitopes of diverse viral envelope glycoproteins can be preferentially recognized by germline-encoded hydrophobic residues at the tip of the HCDR2 loop of IGHV1-69-encoded antibodies. Such class of antibodies can be readily produced by multiple individuals, representing an immunological solution in response to emerging pathogens, and demonstrating an evolutionary advantage for primates to possess the IGHV1-69 gene [36]. However, in the case of SARS-CoV-2, the virus demonstrates a remarkable ability to adapt and evade the host immune response.

Immune evasion by SARS-CoV-2 is highly complex, and other selection pressures could exist for the emergence of L452R. Nevertheless, our analysis points to a link between L452 substitution and herd immunity that is constituted by IGHV1-69-encoded neutralizing antibodies. Insights into antibody-mediated selection pressure on SARS-CoV-2 should provide theoretical framework for better understanding of variant genesis and variant immune evasion.

## Supplementary Material

Supplemental Material
