# Supplementary material for: Shared IGHV1-69-encoded neutralizing antibodies contribute to the emergence of L452R substitution in SARS-CoV-2 variants
Source: Emerg Microbes Infect. 2022 Nov 11;11(1):2749–61. doi: 10.1080/22221751.2022.2140611 (PMC9662066; doi:10.1080/22221751.2022.2140611)
Supplement: Supplemental Material [file TEMI_A_2140611_SM1238.docx]

**
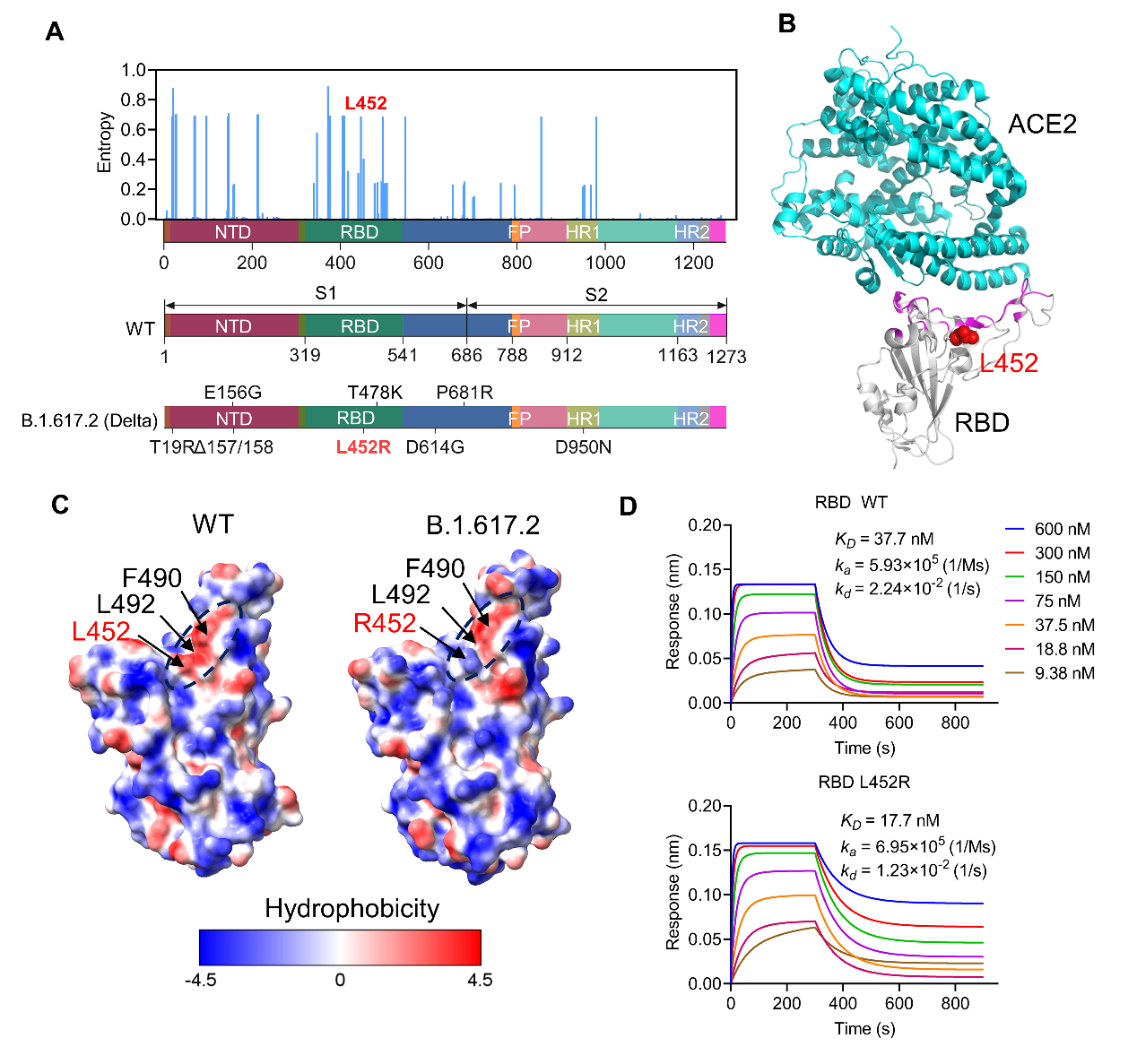
**

**Figure S1. Effects of L452R on characteristics of SARS-CoV-2 RBD.** (**A**) Sequence conservation of each residue on SARS-CoV-2 spike was quantified by sequence entropy. (**B**) Cartoon representation showing the structure of ACE2-RBD complex. Residue L452 was highlighted and labeled. (**C**) Surface representations showing the hydrophobicity of SARS-CoV-2 WT and B.1.617.2 (Delta) RBDs. (**D**) ACE2 binding by WT and L452R RBDs as measured by BLI. ACE2-Fc were immobilized onto protein A biosensors and ACE2-RBD binding was assessed by dipping ACE2-Fc loaded biosensors into indicated RBD dilution series.


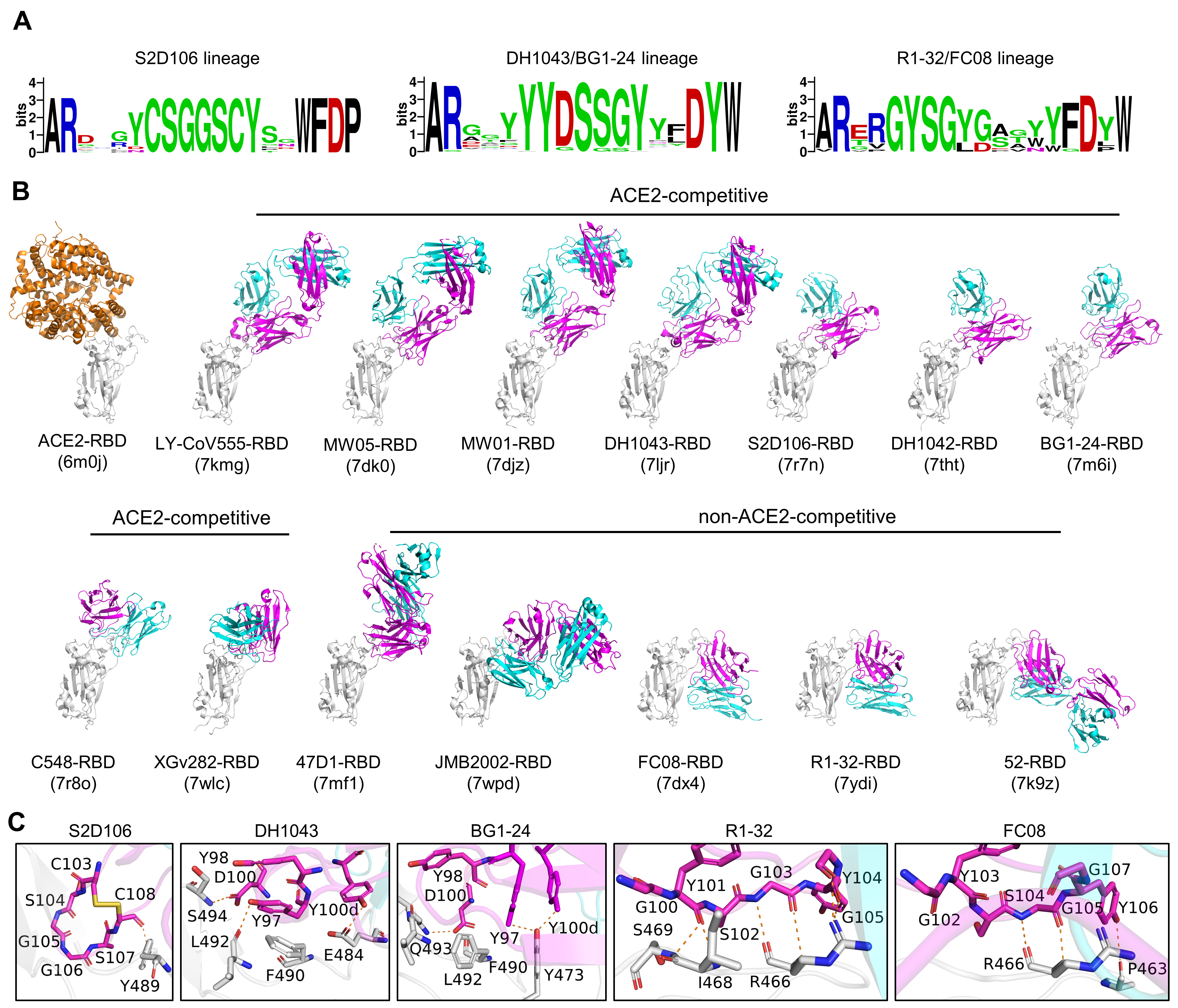


**Figure S2. Sequence and structure analysis of the** **IGHV1-69-encoded L452-contacting mAbs.** (**A**) Sequence logo plot showing the HCDR3s of three shared L452-contacting antibody clonotypes that related to S2D6 (left panel), DH1043/BG1-24 (middle panel), or R1-32/FC08 (right panel). (**B**) Cartoon representations showing the binding mode of the ACE2 or IGHV1-69-encoded L452-contacting mAbs with RBD. RBDs are colored in gray, ACE2 is colored in orange, antibody heavy chains are colored in magenta, and antibody light chain are colored in cyan. (**C**) Interactions between HCDR3 motif of three shared L452-contacting antibody clonotypes and RBD are primarily mediated by hydrophobic contacts. Residues in the RBD are colored in gray, and residues in antibody heavy chain and light chains are colored in magenta and cyan, respectively. Hydrogen bonds are colored in orange.


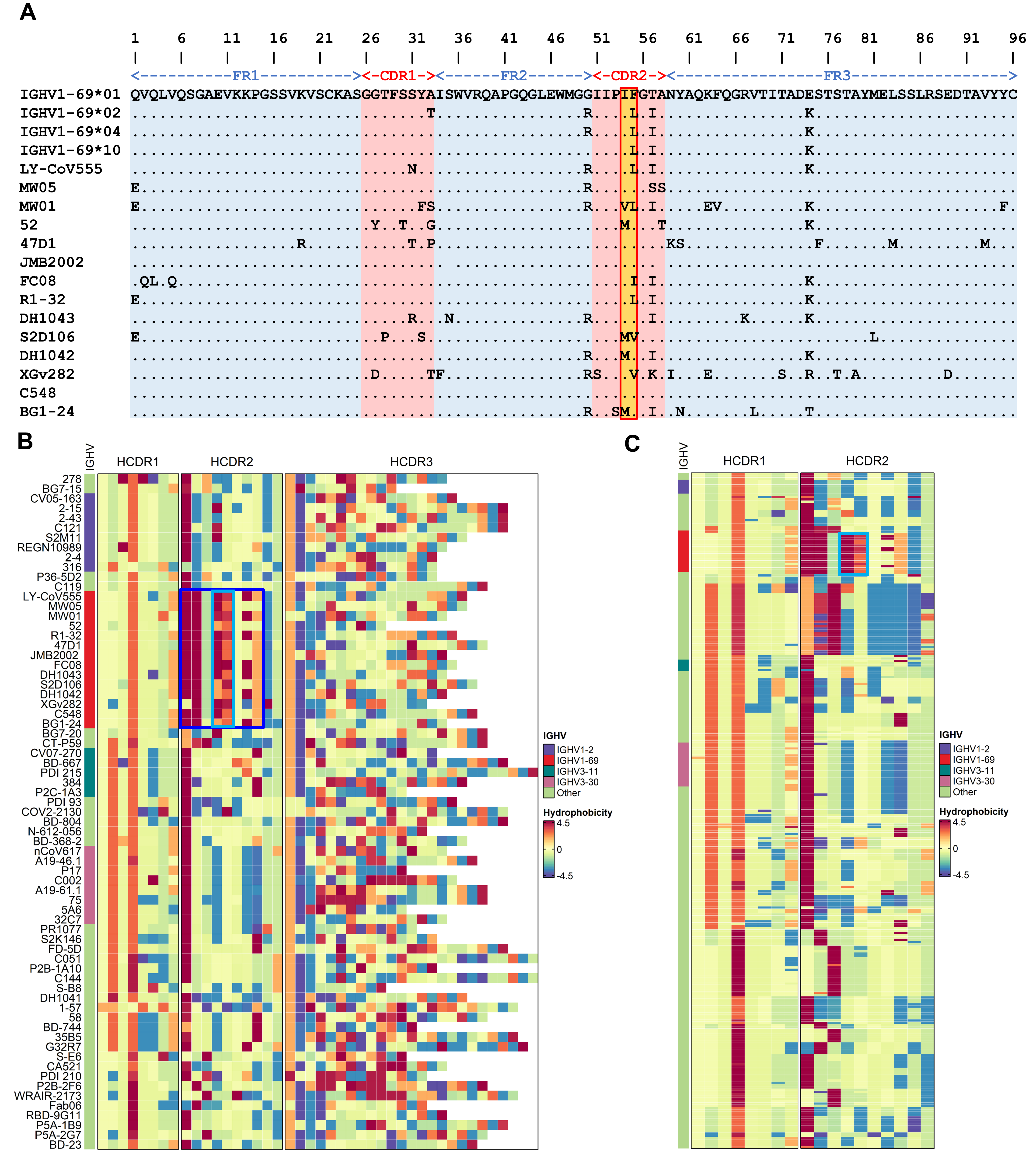


**Figure S3. Genetic and** **hydrophobicity analysis of the** **L452-contacting mAbs.** (**A**) Sequence alignment of IGHV1-69-encoded heavy chains and IGHV1-69 germline sequences. (**B**) Heatmap showing the hydrophobicity of L452-contacting mAbs at HCDR loops. HCDR2 loops of IGHV1-69 antibodies are generally more hydrophobic (highlighted in deep blue box) and they share 2 conserved hydrophobic amino acids at the tip of HCDR2 loop (highlighted in light blue box). (**C**) Heatmap showing the hydrophobicity of all the human IGHV germline genes. IGHV1-69 HCDR2 loop and the tip of HCDR2 loop were highlighted by deep blue and light blue boxes respectively.


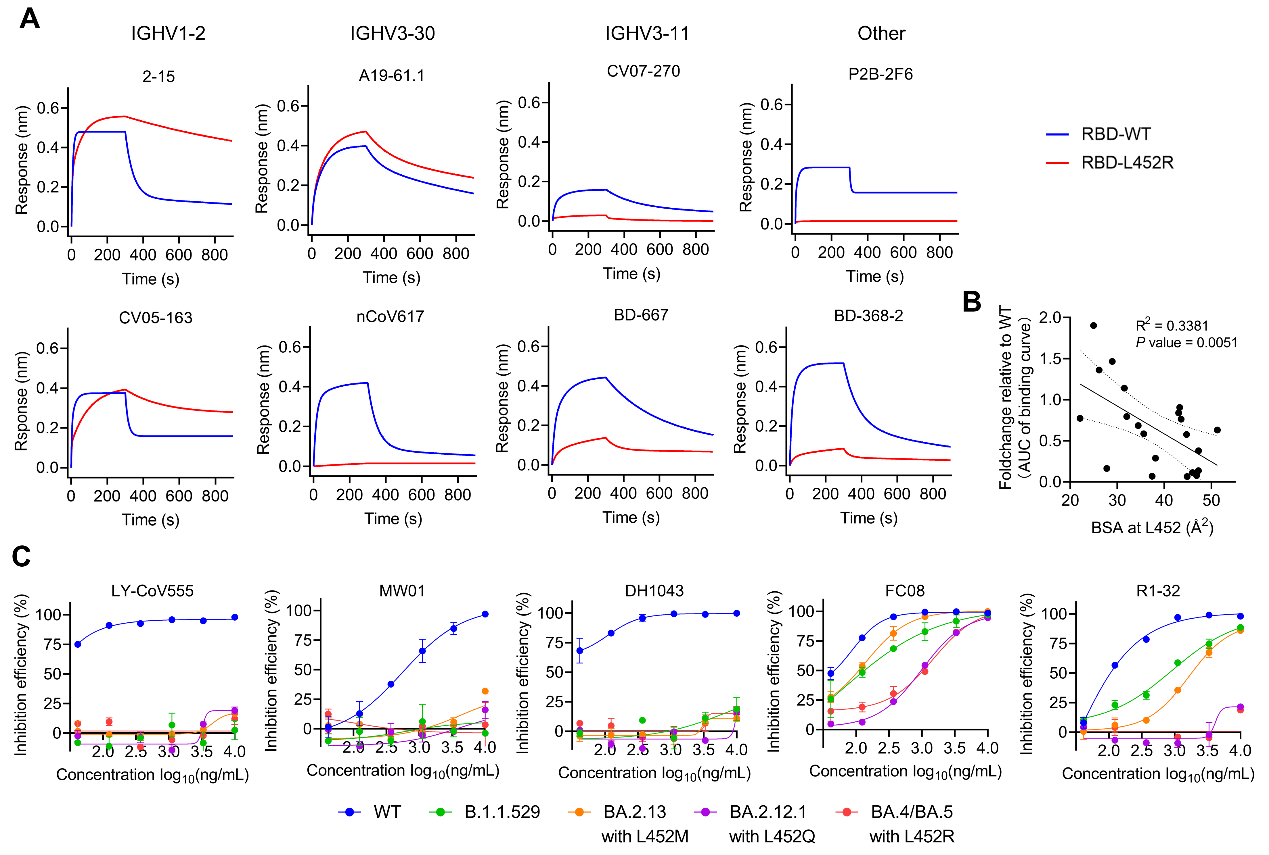


**Figure S4.** **Binding and neutralization activities of L452-contacting mAbs.** (**A**) Binding abilities of IGHV1-2, IGHV3-30, IGHV3-11, and other IGHVs-encoded mAbs to WT or L452R RBDs were assessed by BLI. (**B**) Correlations between antibody binding affinity fold-change to L452R RBD relative to WT RBD and BSA at L452. The fold-change calculated by dividing the area under the curve (AUC) derived from L452R binding curves by the AUC derived from WT binding curves. (**C**) Neutralizing activities of IGHV1-69-encoded mAbs to WT, Omicron (B.1.1529), and Omicron subvariants with L452 substitutions were measured by pseudovirus-neutralization assay.


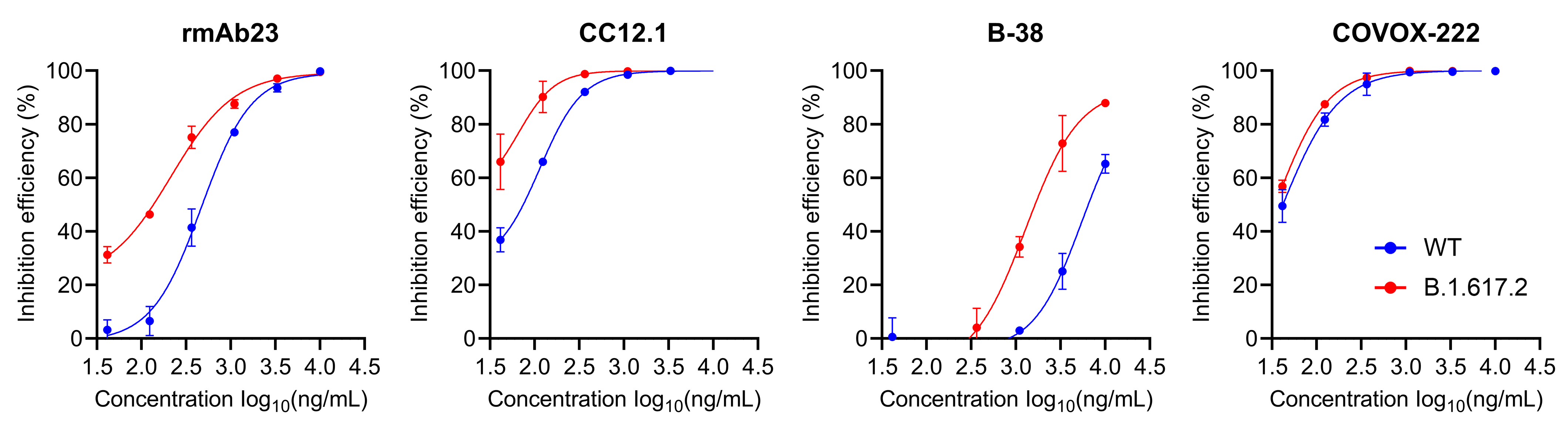


**Figure S5.** **Neutralization activities of four representative IGHV3-53-encoded mAbs to WT and B.1.617.2 (Delta).**

**
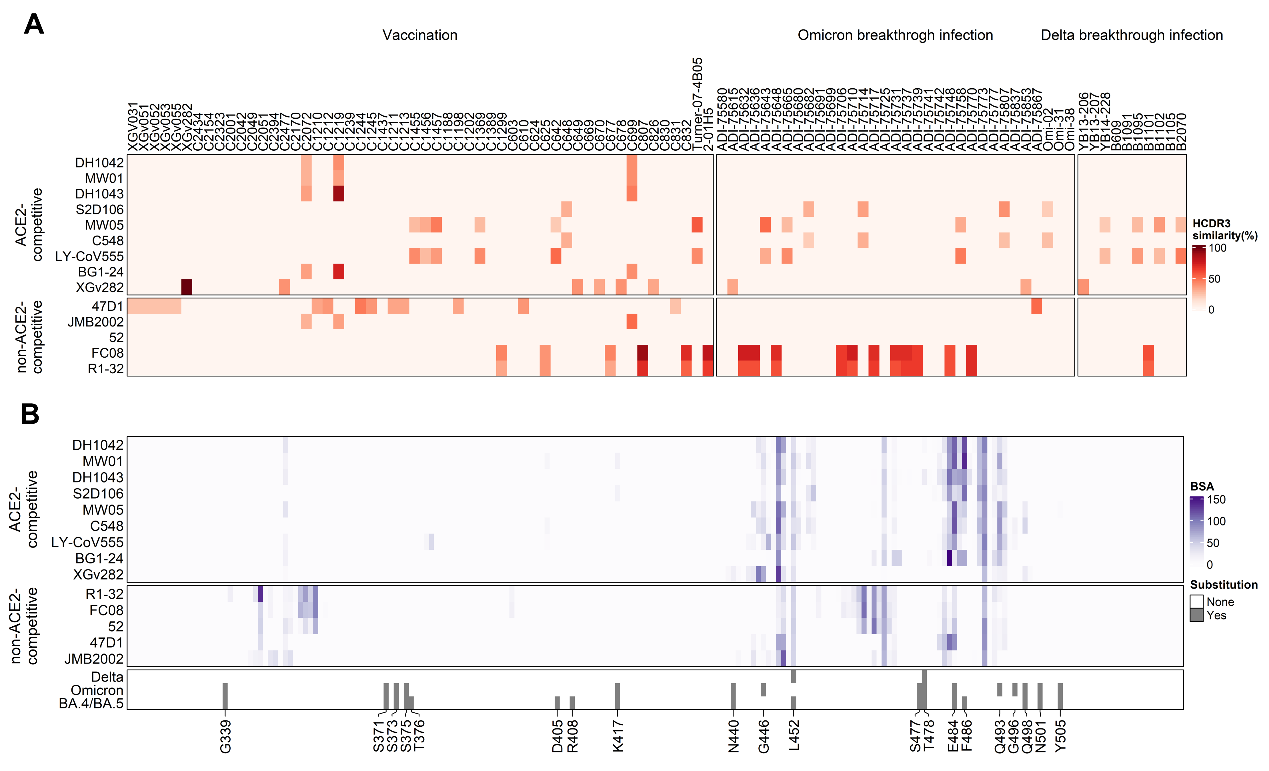
**

**Figure S6. Sequence and epitope analysis of the IGHV1-69-encoded L452-contacting mAbs.** (**A**) Heatmap showing the HCDR3 similarity between 14 structurally characterized IGHV1-69-encoded L452-contacting mAbs and 90 IGHV1-69-encoded RBD mAbs isolated from vaccination, Delta breakthrough infection, or Omicron BA.1 breakthrough infection. (**B**) Heatmap showing the buried surface area (BSA) of epitope residues of 14 structurally characterized IGHV1-69-encoded L452-contacting mAbs. The bottom annotation indicates the mutation sites in Delta, Omicron and BA.4/BA.5 strains.

**Table S1 Summary of the 221 SARS-CoV-2 RBD-specific mAbs.**

| **mAb Id** | **HC germline** | **LC germline** | **PBD Id** | **BSA at L452 (Å2)** |
| --- | --- | --- | --- | --- |
| C102 | IGHV3-53 | IGKV3-20 | 7K8M | 0 |
| C002 | IGHV3-30 | IGKV1-39 | 7K8S | 27.94 |
| C104 | IGHV4-34 | IGKV3-20 | 7K8U | 0 |
| C119 | IGHV1-46 | IGLV2-14 | 7K8W | 29.36 |
| C121 | IGHV1-2 | IGLV2-23 | 7K8X | 14.23 |
| C144 | IGHV3-53 | IGLV2-14 | 7K90 | 4.18 |
| C135 | IGHV3-30 | IGKV1-5 | 7K8Z | 0 |
| C110 | IGHV5-51 | IGKV1-5 | 7K8V | 0 |
| S2H13 | IGHV3-7 | IGLV7-46 | 7JV2 | 0 |
| S2H14 | IGHV3-15 | IGLV6-57 | 7JX3 | 0 |
| S2A4 | IGHV3-7 | IGLV6-57 | 7JVA | 0 |
| S304 | IGHV3-13 | IGKV1-39 | 7JW0 | 0 |
| EY6A | IGHV3-30 | IGKV1-39 | 6ZDH | 0 |
| CT-P59 | IGHV2-70 | IGLV1-51 | 7CM4 | 47.16 |
| CB6 | IGHV3-66 | IGKV1-39 | 7C01 | 0 |
| CV07-250 | IGHV1-18 | IGLV2-8 | 6XKQ | 0 |
| CV07-270 | IGHV3-11 | IGLV2-14 | 6XKP | 46 |
| S309 | IGHV1-18 | IGKV3-20 | 6WPS | 0 |
| P2B-2F6 | IGHV4-38-2 | IGLV2-8 | 7BWJ | 37.4 |
| C105 | IGHV3-53 | IGLV2-8 | 6XCM | 0 |
| CR3022 | IGHV5-51 | IGKV4-1 | 6W41 | 0 |
| B-38 | IGHV3-53 | IGKV1-9 | 7BZ5 | 0 |
| COVA2-04 | IGHV3-53 | IGKV3-20 | 7JMO | 0 |
| COVA2-39 | IGHV3-53 | IGLV2-23 | 7JMP | 0 |
| BD-23 | IGHV7-4-1 | IGKV1-5 | 7BYR | 8.01 |
| REGN10933 | IGHV3-11 | IGKV1-33 | 6XDG | 0 |
| REGN10987 | IGHV3-30 | IGLV2-14 | 6XDG | 0 |
| CC12.1 | IGHV3-53 | IGKV1-9 | 6XC2 | 0 |
| CC12.3 | IGHV3-53 | IGKV3-20 | 6XC4 | 0 |
| P17 | IGHV3-30 | IGKV1-39 | 7CWM | 32.62 |
| BD-368-2 | IGHV3-23 | IGKV2-28 | 7CHH | 27.78 |
| BD-236 | IGHV3-53 | IGKV1-9 | 7CHB | 0 |
| BD-604 | IGHV3-53 | IGKV1-9 | 7CH4 | 0 |
| BD-629 | IGHV3-53 | IGKV3-20 | 7CH5 | 0 |
| C1A-B3 | IGHV3-53 | IGKV1-9 | 7KFW | 0 |
| C1A-F10 | IGHV3-53 | IGKV1-9 | 7KFY | 0 |
| C1A-C2 | IGHV3-53 | IGKV1-9 | 7KFX | 0 |
| C1A-B12 | IGHV3-53 | IGKV1-9 | 7KFV | 0 |
| P2C-1A3 | IGHV3-11 | IGKV1-9 | 7CDJ | 7.71 |
| P2C-1F11 | IGHV3-66 | IGKV3-20 | 7CDI | 0 |
| CV30 | IGHV3-53 | IGKV3-20 | 6XE1 | 0 |
| 253H165L | IGHV1-58 | IGKV3-20 | 7NDB | 0 |
| 316 | IGHV1-2 | IGLV2-8 | 7ND7 | 2.68 |
| 384 | IGHV3-11 | IGKV1-27 | 7ND8 | 28.95 |
| 150 | IGHV3-53 | IGKV1-9 | 7ND5 | 0 |
| 40 | IGHV3-53 | IGKV1-9 | 7ND6 | 0 |
| 253H55L | IGHV1-58 | IGKV3-20 | 7ND9 | 0 |
| 88 | IGHV4-61 | IGLV1-36 | 7ND4 | 0 |
| 269 | IGHV3-53 | IGKV1-9 | 7NEH | 0 |
| 45 | IGHV3-30-3 | IGKV1-33 | 7BEL | 0 |
| 75 | IGHV3-30 | IGKV1-12 | 7BEN | 19.76 |
| 158 | IGHV3-53 | IGKV3-20 | 7BEJ | 0 |
| 253 | IGHV1-58 | IGKV3-20 | 7BEN | 0 |
| 910-30 | IGHV3-53 | IGKV1-33 | 7KS9 | 0 |
| 2-15 | IGHV1-2 | IGLV2-14 | 7L5B | 24.96 |
| 2-43 | IGHV1-2 | IGLV2-14 | 7L56 | 18.4 |
| 2-4 | IGHV1-2 | IGLV2-8 | 6XEY | 6.03 |
| H4 | IGHV1-2 | IGKV2-40 | 7L58 | 0 |
| DH1041 | IGHV3-7 | IGLV1-40 | 7LAA | 37.9 |
| DH1047 | IGHV1-46 | IGKV4-1 | 7LD1 | 0 |
| DH1043 | IGHV1-69 | IGKV3-20 | 7LJR | 35.64 |
| COVA1-16 | IGHV1-46 | IGKV1-33 | 7JMW | 0 |
| S2E12 | IGHV1-58 | IGKV3-20 | 7K45 | 0 |
| S2M11 | IGHV1-2 | IGKV3-20 | 7K43 | 12.54 |
| 2-7 | IGHV2-5 | IGLV2-14 | 7LSS | 0 |
| 1-57 | IGHV3-72 | IGKV3-20 | 7LS9 | 46.81 |
| P4A1 | IGHV3-53 | IGKV1-12 | 7CJF | 0 |
| CV05-163 | IGHV1-2 | IGKV3-11 | 7LOP | 26.15 |
| 15033-7 | IGHV3-23 | IGKV1-39 | 7KLH | 0 |
| 15033 | IGHV3-23 | IGKV1-39 | 7KLG | 0 |
| P5A-3C12 | IGHV2-5 | IGKV4-1 | 7D0B | 0 |
| P5A-3A1 | IGHV3-53 | IGKV3-20 | 7D0C | 0 |
| P5A-1B8 | IGHV3-53 | IGKV1-9 | 7CZR | 0 |
| P5A-2G7 | IGHV4-61 | IGLV2-14 | 7D03 | 23.41 |
| P5A-1B9 | IGHV4-59 | IGKV4-1 | 7CZX | 28.45 |
| P5A-2F11 | IGHV1-8 | IGKV4-1 | 7CZY | 0 |
| P5A-1B6 | IGHV3-30 | IGKV1-33 | 7CZV | 0 |
| P2B-1A1 | IGHV4-59 | IGLV2-14 | 7CZP | 0 |
| P2B-1A10 | IGHV3-53 | IGKV1-33 | 7CZQ | 6.19 |
| P5A-2G9 | IGHV3-33 | IGLV5-37 | 7CZT | 0 |
| LY-CoV488 | IGHV3-53 | IGKV1-33 | 7KMH | 0 |
| LY-CoV555 | IGHV1-69 | IGKV1-39 | 7L3N | 51.29 |
| LY-CoV481 | IGHV3-53 | IGKV1-12 | 7KMI | 0 |
| CV38-142 | IGHV5-51 | IGKV6-12 | 7LM8 | 0 |
| 222 | IGHV3-53 | IGKV3-20 | 7NX6 | 0 |
| FC08 | IGHV1-69 | IGLV1-40 | 7DX4 | 43.03 |
| STE90-C11 | IGHV3-66 | IGKV1-9 | 7B3O | 0 |
| 47D1 | IGHV1-69 | IGLV2-14 | 7MF1 | 43.58 |
| 5A6 | IGHV3-30 | IGKV1-39 | 7KQB | 14.41 |
| 3D11 | IGHV4-34 | IGLV6-57 | 7M7B | 0 |
| CA521 | IGHV4-34 | IGKV3-11 | 7E23 | 37.3 |
| BG7-20 | IGHV1-8 | IGLV1-40 | 7M6H | 19.41 |
| BG7-15 | IGHV1-18 | IGKV3-20 | 7M6G | 13.19 |
| BG10-19 | IGHV5-51 | IGLV1-47 | 7M6E | 0 |
| BG1-24 | IGHV1-69 | IGKV3-20 | 7M6I | 22.09 |
| BG4-25 | IGHV3-53 | IGKV3-20 | 7M6D | 0 |
| S2X259 | IGHV1-69 | IGLV1-40 | 7M7W | 0 |
| S2H97 | IGHV5-51 | IGLV2-14 | 7M7W | 0 |
| 47D11 | IGHV4-59 | IGKV3-15 | 7AKJ | 0 |
| PR1077 | IGHV3-33 | IGKV2-28 | 7DEO | 4.84 |
| PR953 | IGHV1-3 | IGKV1-16 | 7DEU | 0 |
| PR961 | IGHV1-46 | IGKV7-3 | 7DET | 0 |
| LY-CoV1404 | IGHV2-5 | IGLV2-14 | 7MMO | 0 |
| 298 | IGHV1-46 | IGKV4-1 | 7K9Z | 0 |
| 52 | IGHV1-69 | IGKV1-39 | 7K9Z | 46.87 |
| CV2-75 | IGHV4-59 | IGLV3-21 | 7M3I | 0 |
| ab1 | IGHV3-53 | IGKV4-1 | 7MJJ | 0 |
| BD-515 | IGHV3-66 | IGKV1-33 | 7E88 | 0 |
| BD-623 | IGHV3-53 | IGLV2-23 | 7E7Y | 0 |
| BD-508 | IGHV3-53 | IGKV1-39 | 7E86 | 0 |
| P22A-1D1 | IGHV3-53 | IGLV1-40 | 7CHS | 0 |
| P5A-3C8 | IGHV3-53 | IGKV1-9 | 7CHP | 0 |
| P5A-1D2 | IGHV3-53 | IGLV1-40 | 7CHO | 0 |
| MW05 | IGHV1-69 | IGKV3-15 | 7DK0 | 47.27 |
| MW01 | IGHV1-69 | IGKV3-15 | 7DJZ | 47.25 |
| MW06 | IGHV3-23 | IGKV1-17 | 7DPM | 0 |
| R1-32 | IGHV1-69 | IGLV1-40 | 7YDI | 39.15 |
| A23-58.1 | IGHV1-58 | IGKV3-20 | 7LRS | 0 |
| B1-182.1 | IGHV1-58 | IGKV3-20 | 7MLZ | 0 |
| COV2-2196 | IGHV1-58 | IGKV3-20 | 7L7E | 0 |
| REGN10989 | IGHV1-2 | IGLV2-14 | 7M42 | 8.35 |
| REGN10985 | IGHV3-9 | IGLV1-40 | 7M42 | 0 |
| S2L20 | IGHV3-30 | IGKV1-33 | 7N8H | 0 |
| 2B11 | IGHV3-66 | IGLV1-40 | 7E5Y | 0 |
| 278 | IGHV1-18 | IGKV1-39 | 7OR9 | 40.63 |
| S2X35 | IGHV1-18 | IGLV1-40 | 7R6W | 0 |
| S2D106 | IGHV1-69 | IGKV1-39 | 7R7N | 34.46 |
| C099 | IGHV3-53 | IGKV3-20 | 7R8L | 0 |
| C051 | IGHV3-53 | IGLV2-14 | 7R8N | 26.26 |
| C032 | IGHV5-51 | IGLV1-40 | 7R8M | 0 |
| C548 | IGHV1-69 | IGLV9-49 | 7R8O | 31.52 |
| C098 | IGHV3-53 | IGKV3-20 | 7N3I | 0 |
| G32R7 | IGHV3-9 | IGKV1-5 | 7N64 | 18.24 |
| RBD-10D12 | IGHV3-53 | IGKV1-9 | 7E3C | 0 |
| RBD-9G11 | IGHV4-59 | IGKV1-33 | 7E3B | 33.8 |
| S-7B8 | IGHV3-33 | IGKV1-17 | 7E39 | 0 |
| COV2-2130 | IGHV3-15 | IGKV4-1 | 7L7E | 26.1 |
| BD-812 | IGHV5-51 | IGKV1-27 | 7EZV | 0 |
| BD-836 | IGHV1-58 | IGKV3-20 | 7EZV | 0 |
| BD-813 | IGHV3-66 | IGKV1-39 | 7EY0 | 0 |
| BD-744 | IGHV3-9 | IGLV3-21 | 7EY0 | 28.43 |
| BD-667 | IGHV3-11 | IGKV3-15 | 7EY4 | 38.13 |
| BD-771 | IGHV3-15 | IGKV1-27 | 7EY5 | 0 |
| BD-821 | IGHV5-51 | IGKV3-15 | 7EY5 | 0 |
| BD-804 | IGHV3-21 | IGKV1D-8 | 7EYA | 41.68 |
| NT-193 | IGHV4-34 | IGKV1-17 | 7E5O | 0 |
| CV503 | IGHV1-69 | IGLV2-8 | 7LQ7 | 0 |
| 58G6 | IGHV1-58 | IGKV3-20 | 7E3L | 0 |
| 13G9 | IGHV1-58 | IGKV3-20 | 7E3K | 0 |
| S-B8 | IGHV3-66 | IGKV4-1 | 7KN3 | 13.21 |
| S-E6 | IGHV4-31 | IGLV1-44 | 7KN4 | 0.17 |
| C022 | IGHV4-39 | IGKV1-5 | 7RKU | 0 |
| C118 | IGHV3-30 | IGLV4-69 | 7RKS | 0 |
| nCoV617 | IGHV3-30 | IGLV1-44 | 7E3O | 44.84 |
| FD20 | IGHV1-69 | IGLV3-25 | 7CYV | 0 |
| CoV11 | IGHV3-53 | IGKV3-20 | 7S4S | 0 |
| N-612-017 | IGHV3-23 | IGKV1-33 | 7S0C | 45.99 |
| N-612-056 | IGHV3-23 | IGKV1-33 | 7S0B | 0 |
| PDI 222 | IGHV1-58 | IGKV3-20 | 7RR0 | 0 |
| WCSL 129 | IGHV3-23 | IGLV1-44 | 7MZI | 0 |
| WCSL 119 | IGHV1-2 | IGLV1-36 | 7MZH | 0 |
| PDI 96 | IGHV1-46 | IGKV4-1 | 7MZK | 0 |
| PDI 93 | IGHV3-15 | IGKV1-39 | 7MZJ | 37.17 |
| PDI 42 | IGHV3-66 | IGKV1-39 | 7MZG | 0 |
| PDI 37 | IGHV3-66 | IGKV1-5 | 7MZF | 0 |
| PDI 215 | IGHV3-11 | IGKV3-20 | 7MZM | 34.95 |
| PDI 210 | IGHV4-34 | IGKV3-11 | 7MZL | 1.34 |
| PDI 231 | IGHV3-53 | IGKV1-33 | 7MZN | 0 |
| WRAIR-2173 | IGHV4-39 | IGLV1-40 | 7N4J | 35.46 |
| WRAIR-2057 | IGHV5-51 | IGKV1-39 | 7N4I | 0 |
| P5C3 | IGHV1-58 | IGKV3-20 | 7P40 | 0 |
| GH12 | IGHV3-7 | IGLV6-57 | 7D6I | 0 |
| XG005 | IGHV2-5 | IGLV2-14 | 7V26 | 0 |
| XG014 | IGHV5-51 | IGLV1-51 | 7V2A | 0 |
| 2-36 | IGHV4-61 | IGKV3-20 | 7N5H | 0 |
| E4 | IGHV3-53 | IGKV1-9 | 7VMU | 0 |
| ION-300 | IGHV5-51 | IGKV3-15 | 7BNV | 0 |
| ION-360 | IGHV3-66 | IGKV1-39 | 7NP1 | 0 |
| MW07 | IGHV3-7 | IGKV1-NL1 | 7DK2 | 0 |
| C1C-A3 | IGHV3-33 | IGKV3-11 | 7SN2 | 0 |
| S2K146 | IGHV3-43 | IGLV1-44 | 7TAS | 13.72 |
| JMB2002 | IGHV1-69 | IGKV1-33 | 7WPD | 43.27 |
| clone 2 | IGHV1-2 | IGKV7-3 | 7MW5 | 0 |
| clone 6 | IGHV1-46 | IGKV7-3 | 7MW4 | 0 |
| 2G1 | IGHV3-53 | IGLV2-8 | 7X08 | 0 |
| CV07-287 | IGHV1-58 | IGKV3-20 | 7S5R | 0 |
| FI-3A | IGHV3-53 | IGKV1-33 | 7Q0A | 0 |
| FD-11A | IGHV3-33 | IGLV1-40 | 7PQZ | 0 |
| FD-5D | IGHV3-48 | IGKV2-28 | 7PR0 | 15.20 |
| DH1042 | IGHV1-69 | IGKV1-39 | 7THT | 33.78 |
| A5-10 | IGHV3-23 | IGKV1-12 | 7F7E | 0 |
| AB-3467 | IGHV4-59 | IGKV1-9 | 7MSQ | 0 |
| 58 | IGHV3-9 | IGLV3-21 | 7QNY | 31.13 |
| XGv289 | IGHV1-46 | IGLV1-47 | 7WEF | 0 |
| XGv265 | IGHV2-5 | IGLV2-14 | 7WEE | 0 |
| XGv347 | IGHV1-58 | IGKV3-20 | 7WED | 0 |
| XGv282 | IGHV1-69 | IGLV1-44 | 7WLC | 32.03 |
| ADI-55688 | IGHV3-21 | IGLV1-40 | 7U2E | 0 |
| ADG20 | IGHV3-21 | IGLV1-40 | 7U2D | 0 |
| G32Q4 | IGHV3-30 | IGLV1-40 | 7SWP | 0 |
| C98C7 | IGHV3-66 | IGKV3-20 | 7SWO | 0 |
| G32A4 | IGHV1-58 | IGKV3-20 | 7SWN | 0 |
| 10-28 | IGHV3-30 | IGKV1-39 | 7SI2 | 0 |
| 10-40 | IGHV4-39 | IGLV6-57 | 7SD5 | 0 |
| 87G7 | IGHV3-23 | IGKV3-11 | 7R40 | 0 |
| 54042-4 | IGHV2-5 | IGKV1-39 | 7T01 | 0 |
| BD-503 | IGHV3-53 | IGKV1-39 | 7EJY | 0 |
| 32C7 | IGHV3-30 | IGKV4-1 | 7EGN | 10.04 |
| 35B5 | IGHV3-9 | IGKV2-28 | 7E9N | 25.10 |
| A19-46.1 | IGHV3-30 | IGLV8-61 | 7TC9 | 43.25 |
| A19-61.1 | IGHV3-30 | IGKV1-12 | 7TBF | 21.18 |
| Fab06 | IGHV4-4 | IGLV2-14 | 7WPH | 2.18 |
| J08 | IGHV1-69 | IGKV3-11 | 7SBU | 0 |
| CV2.6264 | IGHV1-69 | IGKV1-39 | 7QF1 | 0 |
| CV2.2325 | IGHV3-53 | IGKV1-9 | 7QF0 | 0 |
| CV2.1169 | IGHV1-58 | IGKV3-20 | 7QF7 | 0 |
| GW01 | IGHV3-43 | IGLV1-44 | 7EPX | 0 |
| UT28K | IGHV1-58 | IGKV3-20 | 7X7O | 0 |
| P36-5D2 | IGHV1-3 | IGKV1-5 | 7FAF | 44.24 |
| ZWC6 | IGHV3-30 | IGKV3-11 | 7WWM | 0 |
| ZWD12 | IGHV3-7 | IGKV3-11 | 7WWL | 0 |
